# Interaction between a haptoglobin genetic variant and coronary artery disease (CAD) risk factors on CAD severity in Singaporean Chinese population

**DOI:** 10.1002/mgg3.1450

**Published:** 2020-08-13

**Authors:** Xuling Chang, Rajkumar Dorajoo, Yi Han, Ling Wang, Jianjun Liu, Chiea‐Chuen Khor, Adrian F. Low, Mark Yan‐Yee Chan, Jian‐Min Yuan, Woon‐Puay Koh, Yechiel Friedlander, Chew‐Kiat Heng

**Affiliations:** ^1^ Department of Paediatrics Yong Loo Lin School of Medicine National University of Singapore Singapore Singapore; ^2^ Khoo Teck Puat – National University Children’s Medical Institute National University Health System Singapore Singapore; ^3^ Genome Institute of Singapore Agency for Science, Technology and Research Singapore Singapore; ^4^ Departments of Preventive Medicine and Biochemistry & Molecular Medicine Keck School of Medicine University of Southern California Los Angeles CA USA; ^5^ Department of Medicine Yong Loo Lin School of Medicine National University of Singapore Singapore Singapore; ^6^ Singapore Eye Research Institute Singapore National Eye Centre Singapore Singapore; ^7^ National University Heart Centre National University Health System Singapore Singapore; ^8^ Division of Cancer Control and Population Sciences UPMC Hillman Cancer Center University of Pittsburgh Pittsburgh PA USA; ^9^ Department of Epidemiology Graduate School of Public Health University of Pittsburgh Pittsburgh PA USA; ^10^ Saw Swee Hock School of Public Health National University of Singapore Singapore Singapore; ^11^ Health Systems and Services Research Duke‐NUS Medical School Singapore Singapore Singapore; ^12^ School of Public Health and Community Medicine Hebrew University of Jerusalem Jerusalem Israel

**Keywords:** coronary artery disease severity, gene‐environment interaction, haptoglobin, mortality

## Abstract

**Background:**

Haptoglobin (Hp) is a plasma protein with strong anti‐inflammation and antioxidant activities. Its plasma level is known to be inversely associated with many inflammatory diseases, including cardiovascular diseases. However, the association of *HP* genetic variants with coronary artery disease (CAD) severity/mortality, and how they interact with common CAD risk factors are largely unknown.

**Methods:**

We conducted the analysis in a Singaporean Chinese CAD population with Gensini severity scores (N = 582) and subsequently evaluated the significant findings in an independent cohort with cardiovascular mortality (excluding stroke) as outcome (917 cases and 19,093 controls). CAD risk factors were ascertained from questionnaires, and stenosis information from medical records. Mortality was identified through linkage with the nationwide registry of births and deaths in Singapore. Linear regression analysis between *HP* genetic variant (rs217181) and disease outcome were performed. Interaction analyses were performed by introducing an interaction term in the same regression models.

**Results:**

Although rs217181 was not significantly associated with CAD severity and cardiovascular mortality (excluding stroke) in all subjects, when stratified by hypertension status, hypertensive individuals with the minor T allele have more severe CAD (β = 0.073, SE = 0.030, *p* = 0.015) and non‐hypertensive individuals with the T allele have lower risk for mortality (odds ratio = 0.771 (0.607–0.980), *p* = 0.033).

**Conclusion:**

*HP* genetic variant is not associated with CAD severity and mortality in the general population. However, hypertensive individuals with the rs217181 T allele associated with higher Hp levels had more severe CAD while non‐hypertensive individuals with the same allele had lower risk for mortality in the Chinese population.

## Graphical abstract

*Hp* genetic variant is not associated with CAD severity and mortality in the general population. However, hypertensive individuals with the rs217181 T allele which is associated with higher Hp levels had more severe CAD, while non‐hypertensive individuals with the same allele had lower risk for mortality in the Chinese population.

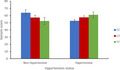

## 1 INTRODUCTION

Haptoglobin (Hp) is an acute phase protein encoded by the *HP* gene (OMIM accession number *140100; Dobryszycka, 1997) and it is present in the plasma in humans (Tseng, Lin, Huang, Liu, & Mao, 2004). One important function of Hp is to capture free hemoglobin (Hb) released during the intravascular destruction of erythrocytes to prevent both iron loss and kidney damage during hemolysis (Kristiansen et al., 2001; MURRAY, CONNELL, & PERT, 1961).

In humans, one of the commonly investigated *HP* variants, rs72294371, is a structural variant which exists in two allelic forms, HP1 and HP2. The HP2 allele has arisen due to the partial duplication of HP1 early in human evolution (Levy et al., 2002; Maeda & Smithies, 1986). The two common alleles result in three *HP* genotypes, HP1‐1, HP2‐1, and HP2‐2 (Cheng et al., 2009; Langlois & Delanghe, 1996). The proteins coded by these three genotypes are functionally distinct. Compared with proteins coded by HP2‐1 and HP2‐2, HP1‐1 protein is a more efficient antioxidant with the strongest binding affinity to Hb (Schaer, Schoedon, Imhof, Kurrer, & Schaer, 2006) and is more rapidly cleared from circulation because of its shorter half‐life (Asleh et al., 2003). Along with the prominent difference in the antioxidative properties of proteins coded by the different *HP* genotypes, Hp has been reported to be associated with many inflammatory diseases, including cardiovascular disease, autoimmune disorders and diabetes (Langlois & Delanghe, 1996; Levy, 2006). Although the impact of this *HP* polymorphism on cardiovascular disease, including risk stratification (Levy et al., 2002), has been investigated in various studies, the results have been controversial and most of the studies were conducted in non‐Chinese population (De Bacquer et al., 2001; Levy et al., 2002; Simpson et al., 2011; Suleiman et al., 2005). Currently, the *HP* rs72294371 polymorphism is generally determined by protein PAGE, PCR and quantitative PCR (Koch et al., 2002; Soejima & Koda, 2008). It has not been successfully evaluated with any array‐based copy number analysis or low‐coverage sequencing rapidly and accurately (Conrad et al., 2010; Consortium GP, 2012), which consequently limits its potential in clinical applications. Recently, with the rapid development of genome‐wide association studies (GWAS), single nucleotide polymorphisms (SNP) tagging the *HP* common variant have been identified and some studies showed that the *HP* common polymorphism can be imputed based on GWAS. A proxy for tagging this variant as reported by a previous study is rs217181 (Boettger et al., 2015).

In this study, we investigated whether the *HP* genetic variant, rs217181, is associated with coronary artery disease (CAD) severity and cardiovascular mortality (excluding stroke) in two Singapore Chinese populations. We also tested for the interactions of this genetic variant with common CAD risk factors, including type 2 diabetes (T2D), hypertension, smoking, and hypercholesterolaemia, for their impact on CAD severity and mortality.

## 2 MATERIALS AND METHODS

### 2.1 Ethical compliance

We performed the analysis with data from two Singapore Chinese datasets, the Singapore Coronary Artery Disease Genetics Study (SCADGENS) and Singapore Chinese Health Study (SCHS). SCADGENS was approved by the National Health Group Domain Specific Review Boards (NHG DSRB). The study procedures were carried out in accordance with the relevant ethics guidelines and regulations. All study participants provided written informed consent. SCHS was approved by the Institutional Review Boards of the National University of Singapore and the University of Minnesota, and all study subjects gave written informed consent.

### 2.2 Study subjects

SCADGENS is an ongoing multiethnic study which commenced in 2011 to assess the genetic determinants of CAD in Singapore. The cohort enrolls patients who undergo diagnostic coronary angiography at the National University Heart Centre of the National University Hospital (NUH). CAD was defined as coronary artery stenosis of at least 50% in one or more epicardial coronary arteries or their branches based on angiography. At recruitment, all study subjects were interviewed face‐to‐face by a research nurse using a standard questionnaire. The questionnaire includes information related to demography, lifestyle, personal medical history, disease history, family disease history, physical activities, and food intake.

SCHS is a long‐term prospective study focused on dietary, genetic and environmental determinants of cancer and other chronic diseases in Singapore (Hankin et al., 2001). Between April 1993 and December 1998, a total of 63,257 Chinese individuals aged 45–74 years were recruited into the study. At recruitment, all the study subjects were interviewed in‐person by a trained interviewer with a structured questionnaire. Since April 1994, a total of 28,439 participants donated blood specimens.

### 2.3 Coronary artery disease severity and mortality

In SCADGENS, CAD history, coronary artery dominance and detailed information about coronary artery stenosis were ascertained through review of medical records. Patients with a history of CAD before the date of recruitment were excluded from the current study. This was to ensure accurate grading of CAD severity based on the first angiographic assessment of fresh cases that did not have any prior procedural interventions.

The Gensini score is a commonly used index to define the CAD severity (Gensini, 1983). It assigns different weights to the arteries depending on the extent of luminal narrowing, vessel size and the geographical importance of their locations. Luminal narrowing is grouped into 1%–25%, 26%–50%, 51%–75%, 76%–90%, 91%–99%, total occlusion and assigned 1, 2, 4, 8, 16, and 32 points, respectively. Vessel size and importance were weighted by a value from 0.5 to 5.0, depending on the amount of myocardium served by the segment. Detailed information for arterial segments included and their weights have been described previously (Gensini, 1983; Ringqvist et al., 1983). The product of these two weights constitutes the total weight for each arterial segment. The Gensini score for each individual was calculated as the sum of the total weights for each segment.

For replication of our findings, we used data from the independent SCHS cohort. However, as information regarding CAD severity is not available for this cohort, we chose cardiovascular deaths as a proxy for severe CAD. In SCHS, cardiovascular deaths from the date of the baseline interview through 31 December 2017 were identified through linkage with the nationwide registry of births and deaths in Singapore. The International Classification of Diseases (ICD) 9th (ICD9; CDC, Last updated 6 Nov 2015. Cited 7 Nov 2019) or 10th (ICD10; CDC, Last updated 15 Apr 2016. Cited 7 Nov 2019; WHO Cited 7 Nov 2019) revision codes were used to classify causes of deaths from cardiovascular diseases [ICD9 (390–459) or ICD10 (I00–I99)] with deaths from cerebrovascular disease excluded.

### 2.4 CAD risk factors

In both SCADGENS and SCHS, self‐reported hypertension status was ascertained from questionnaires. In SCADGENS, systolic blood pressure (SBP) and diastolic blood pressure (DBP) were obtained from medical records. In SCHS, SBP was measured three times with a 3‐min interval between each measurement after the participants were seated at rest for at least 5 min, according to validated standard procedures (Chang et al., 2017; Vera‐Cala, Orostegui, Valencia‐Angel, López, & Bautista, 2011). The average value of the three readings was used for analysis. In both cohorts, participants were classified as hypertensive if they met one of the following criteria: (1) SBP ≥ 140 mm/Hg; (2) DBP ≥90 mm/Hg; (3) self‐reported to have a history of hypertension or on anti‐hypertensive medication. In SCADGENS, T2D and hypercholesterolaemic patients were identified based on their self‐reported disease from questionnaire and medical history. Participants were classified into never‐smokers, ever‐smokers, and current smokers based on their responses to the questions regarding cigarette smoking in the questionnaires.

### 2.5 Genotyping and SNP selection

Chinese CAD patients from SCADGENS included in the current study were cases from a previous GWAS case–control study (Han et al., 2017). Rs217181 was a constituent SNP of the Illumina HumanOmniZhongHua‐8 genotyping array. Detailed descriptions regarding genotyping and quality control (QC) procedures have been published previously (Han et al., 2017). A total of 270 CAD patients genotyped in the first batch who did not have a prior history of CAD (SCADGENS 1) and 312 patients without a CAD history genotyped in the second batch (SCADGENS 2) were included in current study.

In SCHS, 18,114 samples were genotyped on the Illumina Global Screening Array (GSA) v1.0 and 7,159 samples were genotyped on the Illumina Global Screening Array v2.0. Detailed descriptions regarding genotyping and QC procedures have been published before (Dorajoo et al., 2019) and a total of 20,010 individuals with relevant phenotype data were included in the current study.

### 2.6 Statistical analysis

The main demographic and clinical characteristics for the datasets are presented in Table 1 and Table S1 for SCADGENS, and in Table 2 for SCHS. Quantitative variables were presented as mean ± SD (standard deviation) or median (interquartile). A two‐sample t‐test was used for normally distributed variables while the Mann–Whitney U test was used for non‐normally distributed variables. Categorical variables were presented as number of individuals (percentage %) and the Pearson's χ^2^ test was used to compare differences in proportions and for checking significant departure of genotype frequencies from Hardy–Weinberg expectations (HWE). In SCADGENS, linear regression was used to investigate the association between rs217181 SNP and log transformed Gensini score. Age, gender, hypercholesterolaemia, hypertension, T2D, and smoking were included into the model as covariates. Interaction analyses were performed by introducing the interaction term (CAD risk factor x SNP) with the specific CAD risk factor and SNP included as covariates in the same regression model. The results were adjusted for multiple comparisons using Bonferroni correction. Significant results after adjustment were further stratified by the specific CAD risk factor. Since the samples in SCADGEDNS were genotyped in two batches, analysis was carried out in SCADGENS 1 and 2 separately to avoid batch effect, and subsequently meta‐analyzed using the fixed‐effects inverse‐variance weighted method. Cochran's Q test was used to measure heterogeneity of effect and a Q_p_ value cut‐off <0.05 was used to determine between‐study heterogeneity (Zeggini & Ioannidis, 2009). Significant findings were further evaluated in SCHS with cardiovascular mortality (excluding stroke) as outcome. The same analytical method was applied with age and gender as covariates. All statistical analyses were carried out using STATA 15.0 (Stata Corp) and a 5% type I error was set to indicate statistical significance (two‐tailed) in all analyses.

**Table 1:** Characteristics of study subjects in SCADGENS

	SCADGENS
	N = 582
Male (%)	545 (93.64%)
Age (year)	57.37 ± 8.87
Hypertension (%)	442 (75.95%)
Diabetes (%)	177 (30.41%)
Hypercholesterolaemia (%)	410 (70.45%)
Smoking	
Ever (%)	150 (25.77%)
Current (%)	198 (34.02%)
rs217181	
CC	261 (44.85%)
CT	244 (41.92%)
TT	77 (13.23%)
MAF	0.342
Gensini score	49.75 (36.00,69.50)

Data is presented as mean ±standard deviation, N (%) or median (interquartile range).

Abbreviations: MAF, minor allele frequency; SCADGENS, Singapore Coronary Artery Disease Genetics Study.

**Table 2:** Characteristics of study subjects in SCHS

	Case	Control	*p*
	N = 917	N = 19,093	
Male (%)	582 (63.47%)	8,324 (43.60%)	**<0.001**
Age (year)	62.00 (56.00, 67.00)	54.00 (48.00,60.00)	**<0.001**
Hypertension (%)	738 (80.48%)	10718 (56.14%)	**<0.001**
rs217181			
CC	406 (44.27%)	8,319 (43.57%)	0.904
CT	410 (44.71%)	8,613 (45.11%)	
TT	101 (11.01%)	2,161 (11.32%)	
MAF	0.334	0.339	

Data is presented as mean ± standard deviation (SD) N (%) or median (interquartile range). Significant results are highlighted in bold.

Abbreviations: MAF, minor allele frequency; SCHS, Singapore Chinese Health Study.

## 3 RESULTS

As shown in Table S1, SCADGENS 2 had higher proportion of males and a lower proportion of individuals with self‐reported hypercholesterolaemia compared to SCADGENS 1. No significant differences were observed for age, percentage of individuals with hypertension, self‐report T2D, cigarette smoking, Gensini score and rs217181 genotype distribution. The observed genotype frequencies of rs217181 did not depart significantly from HWE in both datasets (*p* = 0.171 and *p* = 0.329, respectively). In SCHS, significant difference was observed for age, gender and hypertensive status between cases and controls (*p* < 0.001, Table 2). The observed genotype frequencies of rs217181 also did not depart significantly from HWE in controls (*p* = 0.336).

**Figure 1:**
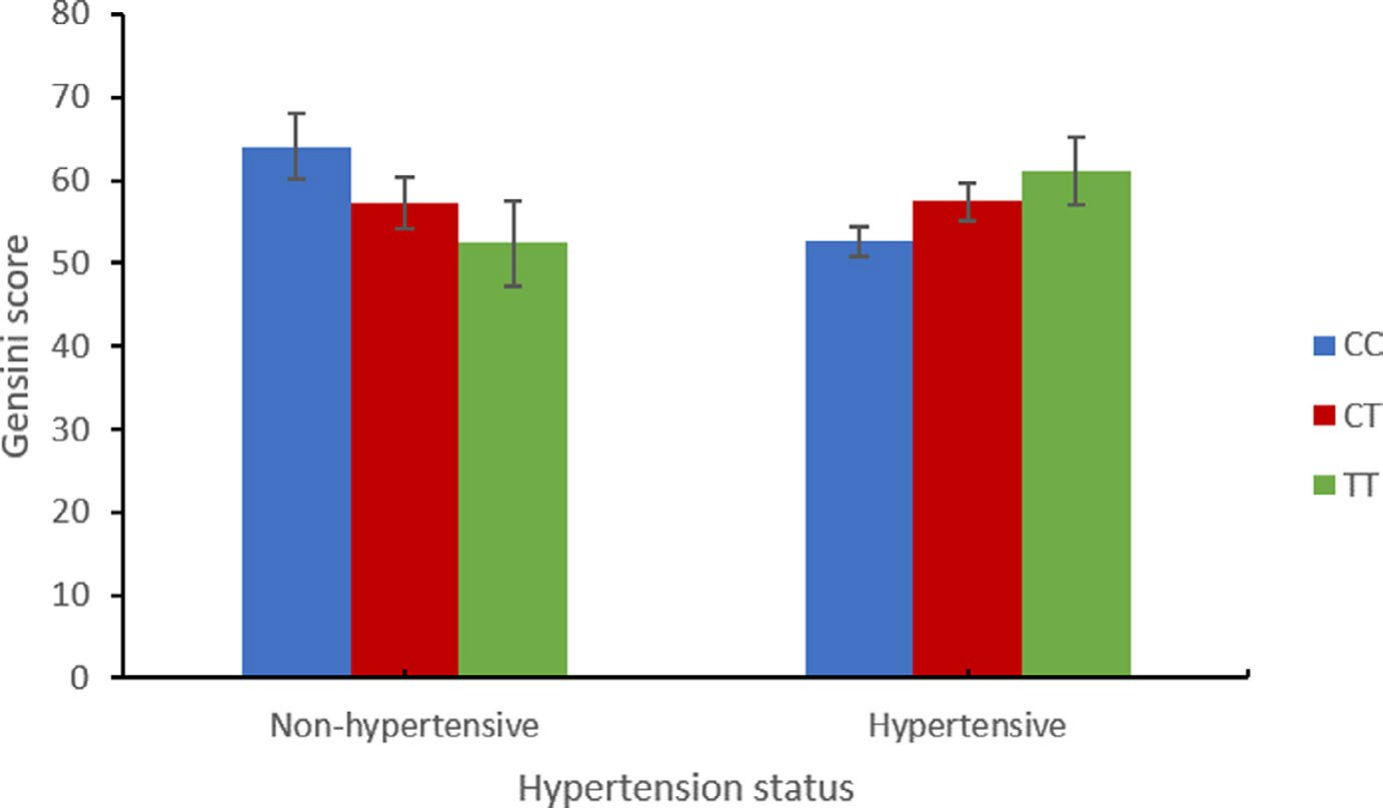
Interaction between rs217181 and hypertension status for coronary artery disease severity in SCADGENS

We first investigated the association between CAD severity (Gensini score) and common CAD risk factors in SCADGENS. Individuals with hypercholesterolaemia had higher Gensini score compared to those without (β = 0.125, SE = 0.042, *p*_adjust_ = 0.011, Table S2). No significant association was observed between Gensini score and the other CAD risk factors (hypertension, T2D, smoking) and the genetic variant rs217181. With inclusion of the CAD risk factor x rs217181 term in the regression models, one significant interaction between the SNP and hypertension on Gensini score after adjustments for multiple tests was observed (β = 0.177, SE = 0.064, *p*_adjust_ = 0.024, Table S3). Stratifying the study subjects by hypertension status showed a positive association between the minor allele T of rs217181 and CAD severity in hypertensive individuals (β = 0.073, SE = 0.030, *p* = 0.015, Table 3, Figure 1, Table S4).

**Table 3:** Interaction effect of haptoglobin variant (rs217181) with hypertension on coronary artery disease severity (Gensini score) in SCADGENS and on cardiovascular deaths (excluding stroke) in SCHS

SCADGENS	Non‐hypertensive			Hypertensive			Interaction	
	N = 140			N = 442				
	Beta	SE	*p*	Beta	SE	*p*	*p*	*p*_adjust_
CAD Severity	−0.097	0.060	0.110	0.073	0.030	**0.015**	**0.006**	**0.024**
SCHS	N = 8,554			N = 11,456				
	OR (95% Cl)		*p*	OR (95% Cl)		*p*	*p*	
Cardiovascular death excluding stroke	0.771 (0.607, 0.980)		**0.033**	1.013 (0.904, 1.134)		0.827	**0.047**	–

Significant results are highlighted in bold.

Abbreviations: Cl, confidence interval; OR, odds ratio; *p*_adjust_, *p* value after adjusting for multiple comparison; SCADGENS, Singapore Coronary Artery Disease Genetics Study; SCHS, Singapore Chinese Health Study.

**Figure 2:**
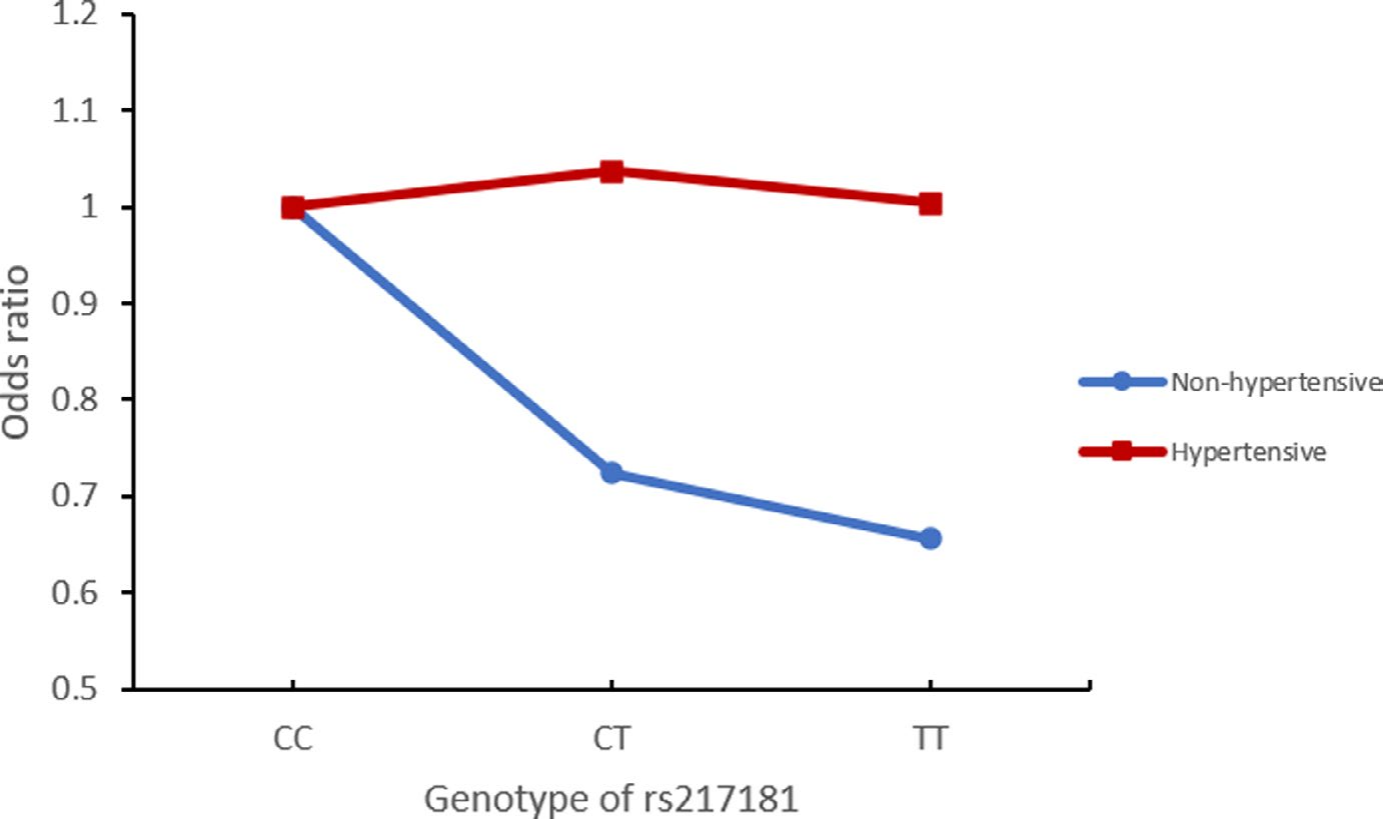
Interaction between rs217181 and hypertension status for coronary death in SCHS

We further evaluated these findings using cardiovascular mortality (excluding stroke) in the SCHS. Hypertensive individuals had 2.3 times higher risk of mortality (*p* < 0.001) while no significant association was observed between mortality and rs217181 (Table S5). When stratified by hypertension status, non‐hypertensive individuals with the minor allele T of rs217181 had significantly lower risk for cardiovascular deaths (excluding stroke; Odds ratio (95% confidence interval) = 0.771 (0.607–0.980), *p* = 0.033, *p*_interaction_ = 0.047, Table 3, Figure 2).

## 4 DISCUSSION

Hp is highly potent in its anti‐inflammatory and antioxidant activities (Purushothaman et al., 2012; Roche, Dantsker, Alayash, & Friedman, 2012). In this study, we investigated the association between the *HP* genetic variant, rs217181, and CAD severity as measured by the Gensini score in a Singaporean Chinese CAD population. We also evaluated whether rs217181 interacts with four common CAD risk factors, hypertension, hypercholesterolaemia, T2D and cigarette smoking to modify the CAD patients’ disease severity. The interaction effect of rs217181 with hypertension on cardiovascular deaths (excluding stroke) was also evaluated in an independent Singapore Chinese population. To the best of our knowledge, our study represents the first investigation on the impact of interactions between *HP* genetic variants and CAD risk factors on CAD severity and mortality in East‐Asians.

Hypertension, hypercholesterolaemia, T2D, and cigarette smoking are established risk factors of CAD (Wilson, 1994). Previous studies have also shown significant association between these risk factors and CAD severity (Hossain, Majumder, Ullah, Shaha, & Mannan, 2015; Larifla et al., 2014; Ockene et al., 1992; Rana et al., 2012; Zeina, Barmeir, Zaid, & Odeh, 2009). In the current study, only hypercholesterolaemia was found to be associated with CAD severity after meta‐analysis and adjusting for multiple testing. We believe the following two reasons may contribute to the inconsistencies. First, although the above four risk factors are well established for CAD and their effects are transferrable between ethnic groups, their effects on CAD severity, on the other hand, have not been as well investigated. Several studies have shown that there are differences between the impact of CAD risk factors on CAD severity and CAD (Escolar, Weigold, Fuisz, & Weissman, 2006; Krishnaswami, Jose, & Joseph, 1994). Second, there are a number of scores and indices that can be used to define CAD severity, such as Gensini (Gensini, 1983), SYNTAX (Sianos et al., 2005), Leaman, Brower, Meester, Serruys, and Van Den Brand (1981), myocardial jeopardy (MJ; Dash, Johnson, Dinsmore, & Harthorne, 1977), and counting the number of diseased vessels (Ringqvist et al., 1983). Most of the previous studies had used the number of diseased vessels to indicate CAD severity, which only considered effects on the three major vessels and a few major sub‐branches. Moreover only a single cut‐off value for stenosis, usually 50%, was used. Based on the stenosis information acquired, we had selected the Gensini score as a measure of CAD severity in our study. Unlike the simpler method of counting the number of vessels diseased, Gensini score also considers the vessel size and its importance, includes the sub‐branches and small vessels, as well as the extent of luminal narrowing by assigning weights accordingly.

Rs217181 was reported to be a proxy SNP for tagging *HP* copy number variant in a European population (Boettger et al., 2015). The minor allele T of rs217181 tags the HP1 allele of the copy number variant. Although HP1 was reported to be a more efficient antioxidant than HP2 (Melamed‐Frank et al., 2001), its effect on cardiovascular disease is controversial, especially in individuals with diabetes (De Bacquer et al., 2001; Levy et al., 2002; Simpson et al., 2011; Suleiman et al., 2005). We did not observe the same association between rs217181 and CAD severity in the subset of diabetic individuals (Table S6). Hypertension was reported to have a complex association with endothelial dysfunction, which precedes the development of adverse cardiovascular events and portends future cardiovascular risk (Dharmashankar & Widlansky, 2010; Shimbo et al., 2010). Previous studies have indicated that CAD is more prevalent and severe in hypertensive patients, especially among individuals with a longer period of hypertension (Zeina et al., 2009). In the current study, hypertension and rs217181 were both not significantly associated with Gensini score. However, when the participants were stratified by their hypertension status, the minor allele T of rs217181 was significantly associated with higher Gensini score only in the hypertensive individuals. The finding was further supported using cardiovascular mortality (excluding stroke) as the outcome in the independent SCHS dataset. Hypertensive individuals have 2.3 times higher risk for mortality in SCHS. Although rs217181 was not associated with mortality in the general population, non‐hypertensive individuals with the minor T allele had much lower risk for mortality when the analysis was stratified by hypertension status. These results indicate that in the Chinese datasets evaluated in the study, haptoglobin genetic variant and a history of hypertension acted interactively to modify CAD severity and mortality.

Recently, a genome‐wide association study (Gurung et al., 2018) identified an East‐Asian specific genetic variant, rs75444904 (upstream of *HP* gene) that can influence urine haptoglobin level. Although rs75444904 is monomorphic in the European populations based on information from the 1000 Genome database, its minor allele frequency (MAF) is between 20 and 30% in the Chinese and Malays. The study also found that rs75444904 is in strong linkage disequilibrium (LD) with the HP allele in East Asians, especially in the Chinese (*r^2^* = 0.869). Thus, the study suggested rs75444904 to be a better surrogate for *HP* structural variant in the East‐Asian Chinese population. In our study, rs75444904 was genotyped on the IlluminaHumanOmniZhongHua‐8 Bead Chip but not on the GSA chip. We also found it to be in strong LD with rs217181 (*r* ^2^ = 0.900). Similar to rs217181, rs75444904 was not associated with CAD severity (Table S2). However, rs75444904 was also observed to significantly interact with hypertension to impact on CAD severity (*p* = 0.031, Table S3). Stratifying the study subjects by hypertension status showed a similar positive association between the minor allele C (plasma Hp increasing allele) of rs75444904 and Gensini score only in hypertensive individuals (*p* = 0.044, Table S4).

Our study has the following two limitations. First, SCADGENS is a study designed to assess the genetic determinants of CAD in Singapore. At enrollment, consented patients who underwent diagnostic coronary angiography were recruited regardless of their prior history of CAD. However, in the current study, only those without prior CAD history were selected. This resulted in the exclusion of a large proportion of patients from the original dataset. Nevertheless, with the present sample size we still had >80% power to detect an interaction effect size of 0.177 (as identified between rs217181 and hypertension in the current study) with MAF = 34% and α = 0.05 in the SCADGENS Chinese samples. In addition, the interaction between the *HP* genetic variant and hypertension was also observed in the larger cohort with cardiovascular deaths (excluding stroke) as the outcome. Second, the Gensini score considered information regarding the extent of luminal narrowing, vessel size and geographical importance of their locations. Compared with Gensini score, SYNTAX score (Sianos et al., 2005) might be able to better reflect and describe CAD severity since it includes additional information for lesion complexity. However, the detailed information needed to construct the SYNTAX score was not readily available for these subjects at the time of analysis.

In conclusion, our study is the first to investigate the association and interaction effect of *HP* genetic variants and common CAD risk factors on CAD severity and cardiovascular mortality (excluding stroke) in the East‐Asian Chinese population. We found that the *HP* genetic variant rs217181 was not associated with CAD severity or mortality in this population. However, when stratified based on hypertension status, hypertensive individuals with the Hp‐elevating minor T allele showed an association with increased CAD severity and non‐hypertensive individuals with this allele showed an association with decreased risk for cardiovascular mortality (excluding stroke).

## CONFLICTS OF INTEREST

The authors declare that they have no competing interests.

## AUTHOR CONTRIBUTIONS

Y.F. and C.‐K.H. conceived and designed the experiments. X.C., Y.H., Y.F. C.‐K. H, W.‐P.K and J.‐M.Y, contributed to the recruitment, sample collection, and data processing. A.F.L. and M.Y.C. ascertained the subjects’ clinical phenotypes. R.D., L.W., J.L., C.‐C.K, W.‐P.K, and J.‐M.Y generated genotyping data. X.C., R.D., Y.H., and C‐K.H. contributed to the statistical and bioinformatics analyses. X.C., R.D., and C‐K.H. drafted the manuscript. All authors critically reviewed the manuscript.

## Supporting information

Table S1‐S6

## Data Availability

The data that supports the findings of this study are available in the supplementary material of this article.
