# Supplementary material for: Interaction between a haptoglobin genetic variant and coronary artery disease (CAD) risk factors on CAD severity in Singaporean Chinese population
Source: Mol Genet Genomic Med. 2020 Aug 13;8(10):e1450. doi: 10.1002/mgg3.1450 (PMC7549588; doi:10.1002/mgg3.1450)
Supplement: Supplementary file 1 — Table S1‐S6 [file MGG3-8-e1450-s001.docx]

**S1 Table.** Characteristics of study subjects in SCADGENS.

|  | SCADGENS 1 | SCADGENS 2 | P |
| --- | --- | --- | --- |
|  | N = 270 | N = 312 |  |
| Male (%) | 244 (90.37%) | 301 (96.47%) | **0.003** |
| Age (year) | 57.73 ± 9.66 | 57.06 ± 8.12 | 0.371 |
| Hypertension (%) | 205 (75.93%) | 237 (75.96%) | 0.992 |
| Diabetes (%) | 87 (32.22%) | 90 (28.85%) | 0.377 |
| Hypercholesterolaemia (%) | 204 (75.56%) | 206 (66.03%) | **0.012** |
| Smoking |  |  |  |
| Ever (%) | 72 (26.67%) | 78 (25.00%) | 0.484 |
| Current (%) | 85 (31.48%) | 113 (36.22%) |  |
| rs217181 |  |  |  |
| CC | 125 (46.30%) | 136 (43.59%) | 0.806 |
| CT | 110 (40.74%) | 134 (42.95%) |  |
| TT | 35 (12.96%) | 42 (13.46%) |  |
| MAF | 0.333 | 0.349 |  |
| Gensini score | 48.00 (35.00,68.00) | 52.50 (36.00,69.75) | 0.339 |

Data is presented as mean ± standard deviation, N (%) or median (interquartile range).

MAF: Minor allele frequency. SCADGENS: Singapore Coronary Artery Disease Genetics Study. Significant results are highlighted in bold.

**S2 Table.** Main association between coronary artery disease severity (Gensini score) and common CAD risk factors/haptoglobin variants in SCADGENS.

|  | SCADGENS 1 | | | SCADGENS 2 | | | Meta-analysis | | | | |
| --- | --- | --- | --- | --- | --- | --- | --- | --- | --- | --- | --- |
|  | N = 270 | | | N = 312 | | | N = 582 | | | | |
|  | beta | se | P | beta | se | P | beta | se | P | P_adjust_ | Q_p-value_ |
| Hypertension | -0.124 | 0.064 | 0.054 | -0.057 | 0.062 | 0.357 | -0.089 | 0.044 | 0.044 | 0.178 | 0.452 |
| Diabetes | 0.045 | 0.060 | 0.451 | 0.047 | 0.058 | 0.418 | 0.046 | 0.042 | 0.268 | 1.000 | 0.986 |
| Hypercholesterolaemia | 0.114 | 0.064 | 0.075 | 0.133 | 0.056 | 0.017 | 0.125 | 0.042 | **0.003** | **0.011** | 0.823 |
| Smoking | 0.099 | 0.033 | 0.003 | -0.014 | 0.029 | 0.624 | 0.034 | 0.022 | 0.117 | 0.470 | **0.010** |
| rs217181 | 0.022 | 0.039 | 0.570 | 0.048 | 0.035 | 0.178 | 0.036 | 0.026 | 0.167 | - | 0.625 |
| rs75444904 | 0.020 | 0.039 | 0.601 | 0.052 | 0.035 | 0.137 | 0.038 | 0.026 | 0.145 | - | 0.544 |

Q: Cochran’s Q, significant Q_p-value_< 0.100 is used to measure between cohort heterogeneity. P_adjust_: P value after adjusting for multiple comparisons. SCADGENS: Singapore Coronary Artery Disease Genetics Study. Significant results are highlighted in bold.

**S3 Table.** Interaction between haptoglobin variants and common coronary artery disease (CAD) risk factors on CAD severity (Gensini score) in SCADGENS.

|  | SCADGENS 1 | | | SCADGENS 2 | | | Meta-analysis | | | | | |
| --- | --- | --- | --- | --- | --- | --- | --- | --- | --- | --- | --- | --- |
|  | N = 270 | | | N = 312 | | | N = 582 | | | | | |
|  | beta | se | P | beta | se | P | beta | se | P | P_adjust_ | Q_p-value_ |  |
| rs217181 |  |  |  |  |  |  |  |  |  |  |  |  |
| Hypertension | 0.178 | 0.089 | **0.046** | 0.175 | 0.091 | 0.057 | 0.177 | 0.064 | **0.006** | **0.024** | 0.978 |  |
| Diabetes | 0.134 | 0.082 | 0.104 | -0.129 | 0.080 | 0.107 | -0.002 | 0.057 | 0.978 | 1.000 | **0.022** |  |
| Hypercholesterolaemia | -0.031 | 0.092 | 0.740 | 0.010 | 0.076 | 0.896 | -0.006 | 0.059 | 0.912 | 1.000 | 0.735 |  |
| Smoking | -0.016 | 0.045 | 0.723 | -0.032 | 0.039 | 0.412 | -0.025 | 0.029 | 0.395 | 1.000 | 0.780 |  |
| rs75444904 |  |  |  |  |  |  |  |  |  |  |  |  |
| Hypertension | 0.105 | 0.092 | 0.254 | 0.169 | 0.089 | 0.059 | 0.138 | 0.064 | **0.031** | - | 0.619 |  |

Q: Cochran’s Q, significant Q_p-value_< 0.100 is used to measure between cohort heterogeneity. P_adjust_: P value after adjusting for multiple comparisons. SCADGENS: Singapore Coronary Artery Disease Genetics Study. Significant results are highlighted in bold.

**S4 Table.** Associations of haptoglobin variants with coronary artery disease severity (Gensini score) by hypertension status in SCADGENS

|  | SCADGENS 1 | | | SCADGENS 2 | | | Meta-Chinese | | | |
| --- | --- | --- | --- | --- | --- | --- | --- | --- | --- | --- |
|  | beta | se | P | beta | se | P | beta | se | P | Q_p-value_ |
| Non-hypertension | N = 65 | | | N = 75 | | | N = 140 | | | |
| rs217181 | -0.103 | 0.085 | 0.233 | -0.090 | 0.086 | 0.296 | -0.097 | 0.060 | 0.110 | -0.103 |
| rs75444904 | -0.051 | 0.091 | 0.574 | -0.092 | 0.083 | 0.270 | -0.074 | 0.061 | 0.228 | 0.740 |
| Hypertension | N = 205 | | | N = 237 | | | N = 442 | | | |
| rs217181 | 0.064 | 0.045 | 0.153 | 0.080 | 0.040 | **0.048** | 0.073 | 0.030 | **0.015** | 0.789 |
| rs75444904 | 0.039 | 0.045 | 0.378 | 0.077 | 0.040 | 0.057 | 0.060 | 0.030 | **0.044** | 0.530 |

Q: Cochran’s Q, significant Q_p-value_< 0.100 is used to measure between cohort heterogeneity. SCADGENS: Singapore Coronary Artery Disease Genetics Study. Significant results are highlighted in bold.

**S5 Table.** Main association between hypertension/rs217181 and coronary artery death in SCHS

|  | N = 20,010 | |
| --- | --- | --- |
|  | Odds Ratio (95%Cl) | P |
| Hypertension | 2.324 (1.962, 2.754) | **< 0.001** |
| rs217181 | 0.974 (0.879, 1.078) | 0.606 |

Cl: Confidence interval. SCHS: Singapore Chinese Health Study. Significant results are highlighted in bold.

**S6 Table.** Associations of haptoglobin genetic variants with coronary artery disease severity (Gensini score) by diabetes status in SCADGENS.

|  | SCADGENS 1 | | | SCADGENS 2 | | | Meta-Chinese | | | |
| --- | --- | --- | --- | --- | --- | --- | --- | --- | --- | --- |
|  | beta | se | P | beta | se | P | beta | se | P | Q_p-value_ |
| Non-diabetic | N = 183 | | | N = 222 | | | N = 405 | | | |
| rs217181 | -0.013 | 0.046 | 0.776 | 0.089 | 0.041 | **0.032** | 0.044 | 0.031 | 0.156 | 0.099 |
| rs75444904 | -0.010 | 0.046 | 0.824 | 0.100 | 0.041 | **0.015** | 0.052 | 0.031 | 0.093 | 0.074 |
| Diabetic | N = 87 | | | N = 90 | | | N = 177 | | | |
| rs217181 | 0.122 | 0.069 | 0.078 | -0.044 | 0.067 | 0.516 | 0.037 | 0.048 | 0.435 | 0.083 |
| rs75444904 | 0.107 | 0.069 | 0.126 | -0.086 | 0.065 | 0.191 | 0.004 | 0.047 | 0.925 | 0.042 |

Q: Cochran’s Q, significant Q_p-value_< 0.100 is used to measure between cohort heterogeneity. SCADGENS: Singapore Coronary Artery Disease Genetics Study. Significant results are highlighted in bold.
